# Investigating the Impact of Different Teaching Methods in College Chinese Courses on Cognitive Understanding and Brain Activation Patterns Using Portable fNIRS

**DOI:** 10.1002/brb3.70934

**Published:** 2025-10-23

**Authors:** Kunpeng Song, Yamei Liu, Peng Xu

**Affiliations:** ^1^ Kaifeng Vocational College of Culture and Arts School of Primary Education Academy Kaifeng China; ^2^ School of Languages and Cultures Shanghai University of Political Science and Law Shanghai China

**Keywords:** brain, Chinese language education, cognition, flipped classroom, fNIRS sensors

## Abstract

**Objective:**

This study aimed to investigate how different instructional methods in college Chinese language education—traditional lecture (TL), group discussion (GD), and flipped classroom (FC)—affect students' cognitive comprehension and prefrontal cortical activation. A key focus was to demonstrate the feasibility and value of using portable functional near‐infrared spectroscopy (fNIRS) sensors to capture real‐time brain activity in ecologically valid classroom settings.

**Methods:**

A total of 30 undergraduate students were randomly assigned to one of three instructional conditions. Participants completed comprehension assessments, a semantic conflict task, and NASA‐TLX workload ratings. Simultaneously, a wearable fNIRS sensor system was used to monitor hemodynamic responses (ΔHbO) and functional connectivity in prefrontal cortical areas, enabling noninvasive neural measurements during naturalistic learning.

**Results:**

The FC condition showed significantly higher total comprehension scores compared to the GD (*p* = 0.002) and TL conditions (*p* < 0.001), with the greatest advantage observed in higher‐order semantic processing (*p* < 0.001). The GD condition outperformed others in resolving semantic conflict, showing the lowest interference cost and highest accuracy in the modern‐meaning interference cost. The FC condition reported the lowest overall cognitive workload (56.7 ± 6.2), significantly lower than GD and TL (*p* < 0.001). fNIRS sensor data revealed the highest ΔHbO activation in the FC condition, particularly in the left DLPFC, along with stronger functional connectivity between L‐DLPFC and L‐VLPFC. Brain–behavior correlations showed a positive association between L‐DLPFC activation and comprehension score (*r* = 0.51, *p* < 0.001), and a negative association between connectivity strength and semantic interference cost (*r* = −0.57, *p* = 0.001).

**Conclusion:**

The integration of portable fNIRS sensors into educational research provides valuable insights into how instructional strategies modulate both cognition and brain function in real‐world classroom environments. The flipped classroom method proves most effective in enhancing semantic comprehension, lowering cognitive demands, and promoting prefrontal engagement. These findings validate the application of wearable brain‐sensing technologies to evaluate and optimize pedagogical practices in higher education.

## Graphical abstract

This study demonstrates that flipped classroom teaching enhances Chinese language comprehension, reduces cognitive load, and increases prefrontal activation and connectivity, as measured by portable fNIRS in a real classroom setting.

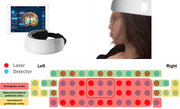

## 1 Introduction

As a core course in China's general education system, college Chinese plays an irreplaceable role in cultivating humanistic literacy, critical thinking, and language expression skills (Feng and Jia 2024). However, current college Chinese instruction still primarily relies on one‐way, lecture‐based teaching methods, which exhibit limitations in complex text analysis (such as intertextual interpretation of classical literature) and the cultivation of higher‐order thinking skills (such as cross‐cultural comparison) (Schindler et al. 2017). Data show that students' engagement in deep semantic processing in traditional classrooms is less than 42%, and the average attention span in class is only 15 min (Froud et al. 2024). Although educational reforms have introduced new models such as flipped classrooms (FCs) and group discussions (GDs), their cognitive neural mechanisms remain unclear—particularly how different teaching methods modulate prefrontal cortex activation patterns to enhance language comprehension, which has become a critical issue for educational neuroscience to address (Immordino‐Yang and Gotlieb 2017).

Existing studies on teaching effectiveness primarily rely on behavioral indicators (such as test scores) and lack real‐time monitoring of cognitive load and brain functional activity (Gruzelier 2014). Traditional neuroimaging techniques like fMRI are difficult to apply in real classrooms due to their spatial confinement and sensitivity to motion artifacts (Pinti et al. 2019; Eastmond et al. 2022). While a few studies using EEG have captured the effects of teaching methods on θ/β oscillations, their spatial resolution is insufficient to precisely locate the functional differentiation of prefrontal subregions (such as the dorsolateral prefrontal cortex [DLPFC] and the ventrolateral prefrontal cortex [VLPFC]) (Putman et al. 2014). This “cognitive black box” state severely limits the scientific basis for precision teaching design.

Breakthroughs in portable functional near‐infrared spectroscopy (fNIRS) technology offer a new solution to this dilemma. Compared to fMRI, next‐generation fNIRS sensors (such as NIRSport2) use multiwavelength laser diodes (690–850 nm) and flexible probe arrays to monitor cortical hemodynamics with 5‐mm spatial resolution under natural head movement (Yücel et al. 2021; Herold et al. 2018). Its core advantages lie in: (1) precise targeting of key brain regions related to language learning—the left DLPFC (L‐DLPFC) is responsible for working memory integration, while the left VLPFC (L‐VLPFC) regulates semantic conflict resolution (Baddeley 2012); (2) temporal resolution (0.1 Hz) sufficient to capture rapid neural responses during instructional interactions (e.g., changes in blood oxygen signals within 7–15 s after a teacher's question). These characteristics make it an ideal tool for classroom neuroeducation research (Pinti et al. 2020).

This study aims to systematically compare the impact of three different teaching methods—traditional lecture (TL), GD, and FC—on students' cognitive understanding and brain activation. Specifically, we examine:

- Differences in cognitive performance across teaching conditions via comprehension tests and semantic conflict tasks.
- Patterns of neural activation in prefrontal brain regions during learning.
- Correlations between brain activation intensity and cognitive test scores.

*Theoretical implications* include advancing our understanding of the neurocognitive mechanisms of effective teaching strategies. *Practical contributions* include providing empirical guidance for instructional design in higher education, supporting personalized teaching, and furthering the development of educational neuroscience.

## 2 Methods

### 2.1 Participants

Thirty undergraduate students (15 females and 15 males; age range: 18–22 years, *M* = 19.4, SD = 0.7) were recruited from the same class of a university‐level Chinese course to ensure consistency in curricular content. All participants had previously enrolled in the course and had no prior exposure to the study materials used in this experiment. A within‐subject design was employed, in which all participants experienced each of the three instructional methods. The sample size was determined based on power analysis and standard fNIRS design practices, ensuring at least 26 valid datasets per condition after potential attrition. Written informed consent was obtained from all participants prior to the experiment, and the study protocol was approved by Shanghai University of Political Science and Law's ethics review board (Approval ID: 10277SPSL2025).

### 2.2 Experimental Design and Procedure

This study employed a within‐subject design with a counterbalanced order of instructional methods across participants to minimize sequence effects. Each participant underwent three instructional modules on classical Chinese vocabulary learning, each employing a distinct pedagogical method: TL, GD, and FC. To rigorously minimize sequence and content effects, the order of the three instructional methods (TL, GD, and FC) and the assignment of the three content units to these methods were fully randomized for each participant. To ensure comparability across modules, the full curriculum unit on classical word understanding was divided into three independents but difficulty‐matched subunits (Units A, B, and C), each containing equivalent semantic complexity and core concepts.

Prior to the instructional sessions, participants completed a brief background questionnaire assessing age, gender, major, previous Chinese course grades, and self‐rated interest in Chinese literature. A short training session was conducted to familiarize participants with the fNIRS device, which included a guided demonstration and a 5‐min resting‐state wearing practice to ensure comfort and reduce novelty‐related motion artifacts during the experimental task.

**FIGURE 1:**
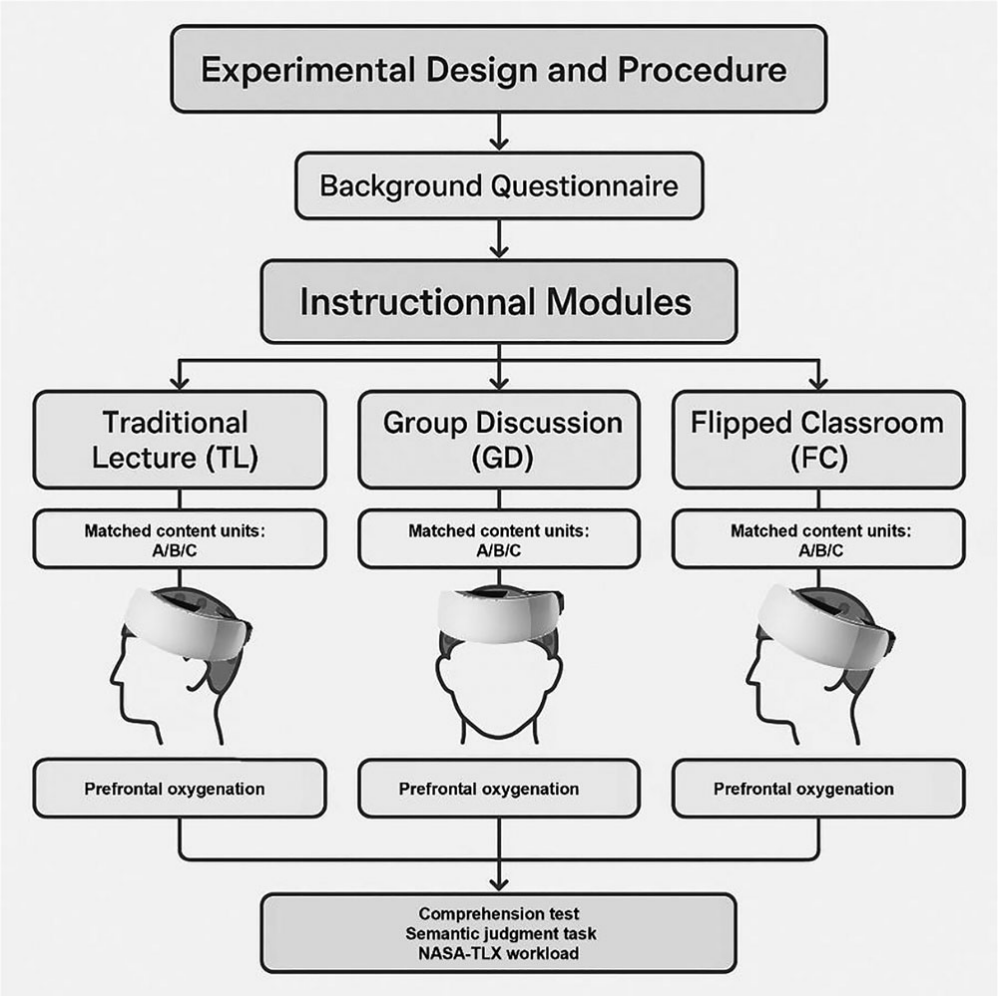
Study design and flowchart.

Each instructional module lasted approximately 40 min and consisted of active classroom teaching followed by immediate assessment. During the session, participants continuously wore the fNIRS cap, which recorded hemodynamic responses in prefrontal brain regions associated with language processing and executive control. After each session, the device was removed, and participants completed a set of post‐instruction tasks, including a comprehension test, a semantic judgment task, and a subjective workload questionnaire (Figure 1).

### 2.3 Cognitive and Behavioral Measures

#### 2.3.1 Immediate Cognitive Understanding Tests

To evaluate students' comprehension following each instructional method, a customized assessment was administered immediately after the session. Each test included a balanced set of items designed to capture varying depths of cognitive processing. First, a set of surface‐level items focused on basic semantic understanding and paraphrasing of classical vocabulary (e.g., accurately interpreting the contextual meaning of archaic words and summarizing their use in classical texts). Second, application‐oriented tasks required students to infer meaning in novel textual contexts, justify word usage in reconstructed sentences, or analyze stylistic implications. Finally, high‐order semantic discrimination questions probed the participants' ability to distinguish subtle differences between classical synonyms or polysemous terms in critical reading passages. Each component was scored separately on a 100‐point scale, and total comprehension scores were computed to reflect overall performance.

#### 2.3.2 Classical Word Semantic Conflict Task

The Word Semantic Interference Task (AWSIT) aims to evaluate the cognitive processing efficiency of participants' deep understanding of classical Chinese vocabulary by measuring their ability to inhibit semantic conflicts in classical words, with a focus on behavioral performance (reaction time and accuracy) when suppressing dominant modern semantic interference. The task adopts a 2 (Judgment Type: Yes/No) × 4 (Condition Type) mixed experimental design, where Condition Type serves as the key independent variable, including four conditions: True Congruent (TC), False Incongruent (FI), False Unrelated (FU), and False Phonographic (FP). In terms of stimulus materials, a target word pool consisting of 40 high‐frequency classical words with ancient‐modern semantic differences (such as zǒu, qù, tì, gòu, kělián, jīn, hé, qīzǐ, xīshēng) was selected, and word frequency and familiarity were balanced through pretests; definition types were respectively matched with authoritative definitions from the Ancient Chinese Dictionary, high‐frequency modern meanings from the Modern Chinese Dictionary, unrelated verbs/nouns, and ancient definitions of orthographically/phonetically similar characters according to different conditions. Each target word appeared once in the four conditions, forming a total of 160 trials, with condition orders pseudorandomized. In the experimental procedure, a single trial sequence included a 500‐ms fixation point “+,” simultaneous presentation of the target word and definition (up to 2000 ms until the participant's response), and a 500‐ms blank screen. Participants were required to judge whether the definition was a common ancient meaning of the word, responding by pressing the F key (left, for “Yes”) or J key (right, for “No”), with emphasis on both speed and accuracy. The experiment consisted of an 8‐trial practice phase with feedback and a formal experiment divided into four blocks with 30‐s breaks between blocks. Behavioral indicators took reaction time and accuracy as core‐dependent variables. The cost of conflict inhibition (FI_RT—FU_RT) and the cost of orthographic‐phonological interference (FP_RT—FU_RT) were calculated to reflect the costs of suppressing modern semantic interference and orthographic/phonological interference, respectively.

#### 2.3.3 Subjective Cognitive Load Assessment

Immediately after each learning module, participants completed a version of the NASA Task Load Index (NASA‐TLX) to report their perceived cognitive workload during the session. The adapted scale comprised six dimensions: mental demands, physical demands, temporal demands, perceived performance, effort, and frustration. Each item was rated on a 0–100 scale in increments of 5, with higher scores indicating greater intensity of experience. The instrument was administered electronically via tablet, and instructions emphasized honest self‐assessment based on the most recent learning experience. Total workload scores were computed as a weighted average of the six components, following standard NASA‐TLX guidelines.

### 2.4 fNIRS Acquisition and Preprocessing

**FIGURE 2:**
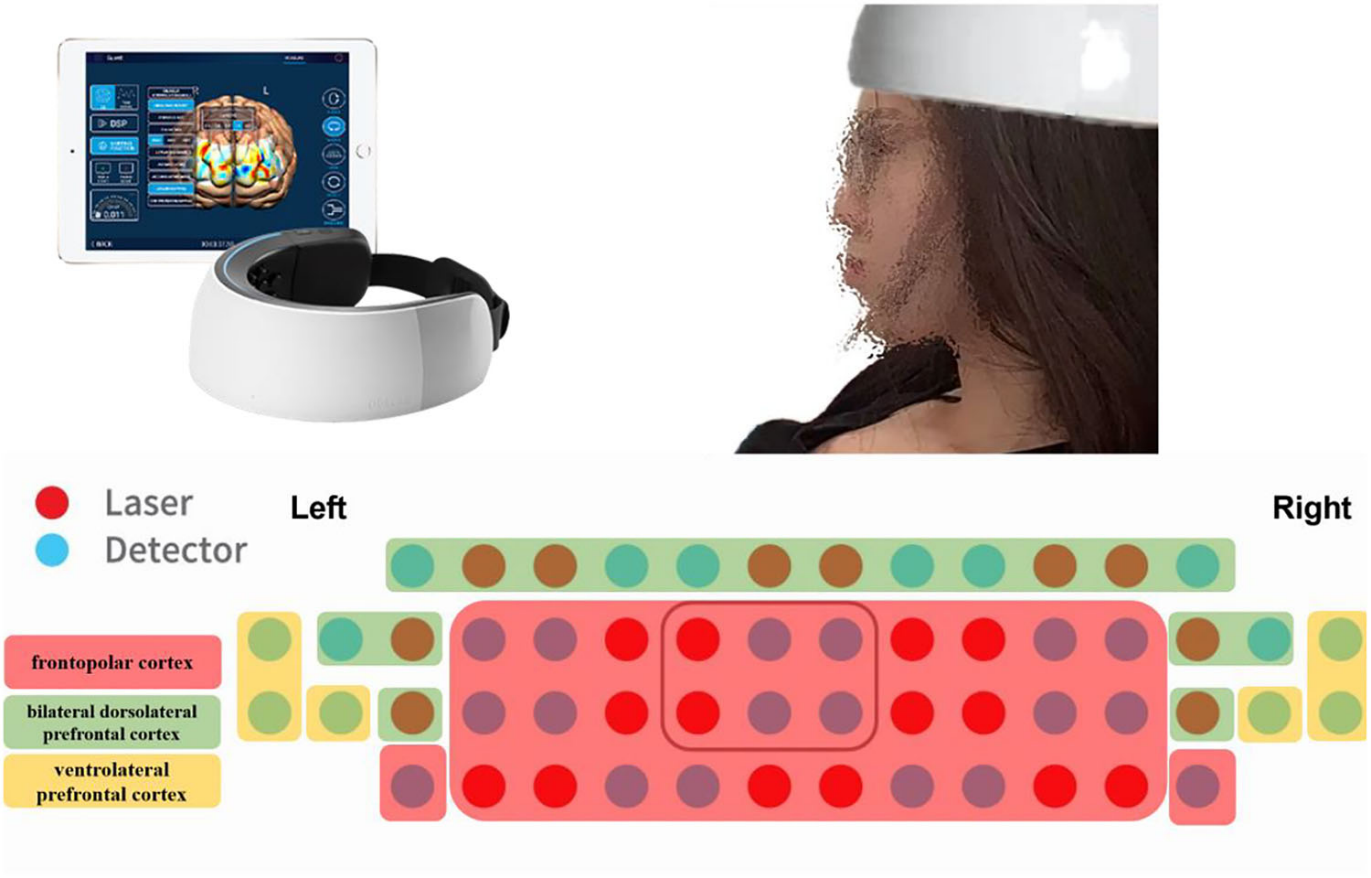
Schematic illustration of the NIRSIT system and prefrontal cortex ROIs.

fNIRS data were recorded continuously throughout the instructional modules using the NIRSIT system (OBELAB Inc., Seoul, Korea), a wearable high‐density optical imaging device with 48 channels targeting prefrontal cortical regions. The device recorded changes in both oxygenated (HbO) and deoxygenated hemoglobin (HbR) concentrations at a sampling rate of 8.138 Hz. The regions of interest (ROIs) focused on bilateral DLPFC, VLPFC, and frontopolar cortex (FPC), areas implicated in executive control, working memory, and semantic regulation (Figure 2).

Preprocessing of the fNIRS data was conducted using MATLAB and Homer2 tools. Motion artifacts were corrected using spline interpolation and wavelet filtering techniques. The raw optical intensity signals were converted into relative concentration changes in HbO and HbR using the modified Beer–Lambert law. A bandpass filter (0.01–0.2 Hz) was applied to remove physiological noise and slow signal drift. For each instructional condition, average ΔHbO values were computed for the entire duration of the lesson and compared across ROIs. In addition, interregional functional connectivity analyses were performed using Pearson correlation coefficients between time‐series signals of DLPFC and VLPFC.

### 2.5 Statistical Analysis

Statistical analyses were conducted using SPSS 26.0. Repeated‐measures ANOVAs were used to examine the effects of instructional methods (TL, GD, and FC) on behavioral performance (comprehension test scores, semantic task accuracy, and RTs), subjective workload ratings, and fNIRS‐derived hemodynamic measures. Sphericity was tested using Mauchly's test, and the Greenhouse–Geisser correction was applied when the assumption was violated. Post hoc pairwise comparisons were Bonferroni‐corrected. Effect sizes were reported as partial eta‐squared (*η* ^2^_p_). Finally, Pearson correlations were calculated between neural activation in target ROIs and behavioral performance metrics to explore potential neurocognitive relationships under different instructional methods. Statistical significance was set at *p* < 0.05 for all tests.

## 3 Results

### 3.1 Behavioral Performance

A repeated‐measures ANOVA revealed a significant main effect of instructional condition on the total score of the immediate comprehension test (*F* (2, 58) = 18.37, *p* < 0.001, *η* ^2^_p_ = 0.39). Bonferroni post hoc tests showed that the FC condition yielded the highest mean score (80.4 ± 5.7), significantly outperforming both the TL condition (65.3 ± 7.8; *p* < 0.001) and the GD condition (73.0 ± 6.9; *p* = 0.002). The GD condition also led to significantly higher scores than the TL condition (*p* = 0.003). Sub‐dimension analyses revealed consistent effects across all three cognitive domains: basic explanation (*F* (2, 58) = 15.29, *p* < 0.001, *η* ^2^_p_ = 0.35), applied analysis (*F* (2, 58) = 17.84, *p* < 0.001, *η* ^2^_p_ = 0.38), and higher‐order semantics (*F* (2, 58) = 21.06, *p* < 0.001, *η* ^2^_p_ = 0.42), with the greatest difference observed in the higher‐order semantic dimension (Table 1).

**TABLE 1:** Comprehension test performance (total score = mean of three subscales).

Measure	TL	GD	FC	*F* (2, 58)	*p*	*η* ^2^_p_
Basic paraphrasing	72.3 ± 8.1	75.6 ± 7.3	82.4 ± 6.5	15.29	< 0.001	0.35
Applied analysis	65.1 ± 9.7	73.5 ± 8.9	80.2 ± 7.4	17.84	< 0.001	0.38
Higher‐order semantics	58.4 ± 11.2	69.8 ± 10.5	78.6 ± 8.3	21.06	< 0.001	0.42
**Total score**	65.3 ± 7.8	73.0 ± 6.9	80.4 ± 5.7	18.37	< 0.001	0.39

For the semantic conflict task, instructional method significantly impacted modern‐meaning interference cost (FI ΔRT: *F* (2, 58) = 12.45, *p* < 0.001, *η* ^2^_p_ = 0.30). The GD condition showed the lowest interference cost (152 ± 38 ms), significantly outperforming TL (218 ± 45 ms; *p* < 0.001) and FC (183 ± 41 ms; *p* = 0.013). Accuracy analysis revealed condition differences in FI trials (*F* (2, 58) = 15.28, *p* < 0.001, *η* ^2^_p_ = 0.35), with GD achieving the highest accuracy (86.2 ± 6.7%), surpassing both TL (74.6 ± 8.3%; *p* < 0.001) and FC (80.1 ± 7.5%; *p* = 0.029) (Table 2).

**TABLE 2:** Semantic conflict task performance.

Index	TL	GD	FC	*F* (2, 58)	*p*	*η* ^2^_p_
Reaction time (ms)						
FI interference cost (Modern‐meaning interference cost)	218 ± 45	152 ± 38	183 ± 41	12.45	< 0.001	0.30
FP interference cost (Phonological interference cost)	197 ± 32	185 ± 29	189 ± 31	1.78	0.178	0.06
Accuracy (%)						
FI condition (Modern‐meaning interference)	74.6 ± 8.3	86.2 ± 6.7	80.1 ± 7.5	15.28	< 0.001	0.35
FU condition (Phonological interference)	92.1 ± 4.8	94.5 ± 3.9	93.3 ± 4.2	2.01	0.143	0.07

NASA‐TLX workload analysis indicated a significant effect of instructional condition on overall perceived workload (*F* (2, 58) = 20.16, *p* < 0.001, *η* ^2^_p_ = 0.41), with the FC condition reporting the lowest workload (56.7 ± 6.2), significantly lower than the TL (71.5 ± 8.9; *p* < 0.001) and GD conditions (65.5 ± 7.4; *p* = 0.002). Dimension‐wise, significant effects were observed in mental demand (*F* (2, 58) = 18.29, *p* < 0.001), time pressure (*F* (2, 58) = 6.32, *p* = 0.003), frustration (*F* (2, 58) = 22.15, *p* < 0.001), physical demand (*F* (2, 58) = 4.87, *p* = 0.011), and effort (*F* (2,58) = 9.43, *p* < 0.001), but not in perceived performance (*p* = 0.215) (Table 3).

**TABLE 3:** NASA‐TLX six‐dimensional workload analysis.

Dimension	TL	GD	FC	*F* (2, 58)	*p*	*η* ^2^_p_
Mental demand	78.2 ± 12.4	72.5 ± 10.8	62.3 ± 9.6	18.29	< 0.001	0.39
Physical demand	41.6 ± 8.3	38.2 ± 7.1	35.7 ± 6.8	4.87	0.011	0.14
Temporal demand	65.4 ± 11.7	68.3 ± 10.2	59.1 ± 8.9	6.32	0.003	0.18
Performance	53.7 ± 9.2	58.4 ± 8.7	61.5 ± 7.3	1.58	0.215	0.05
Effort	81.5 ± 10.6	74.8 ± 9.4	67.3 ± 8.1	9.43	< 0.001	0.25
Frustration	70.8 ± 13.1	55.6 ± 12.3	48.2 ± 10.7	22.15	< 0.001	0.43
**Total workload**	71.5 ± 8.9	65.5 ± 7.4	56.7 ± 6.2	20.16	< 0.001	0.41

### 3.2 Neural Activation Results (fNIRS)

**FIGURE 3:**
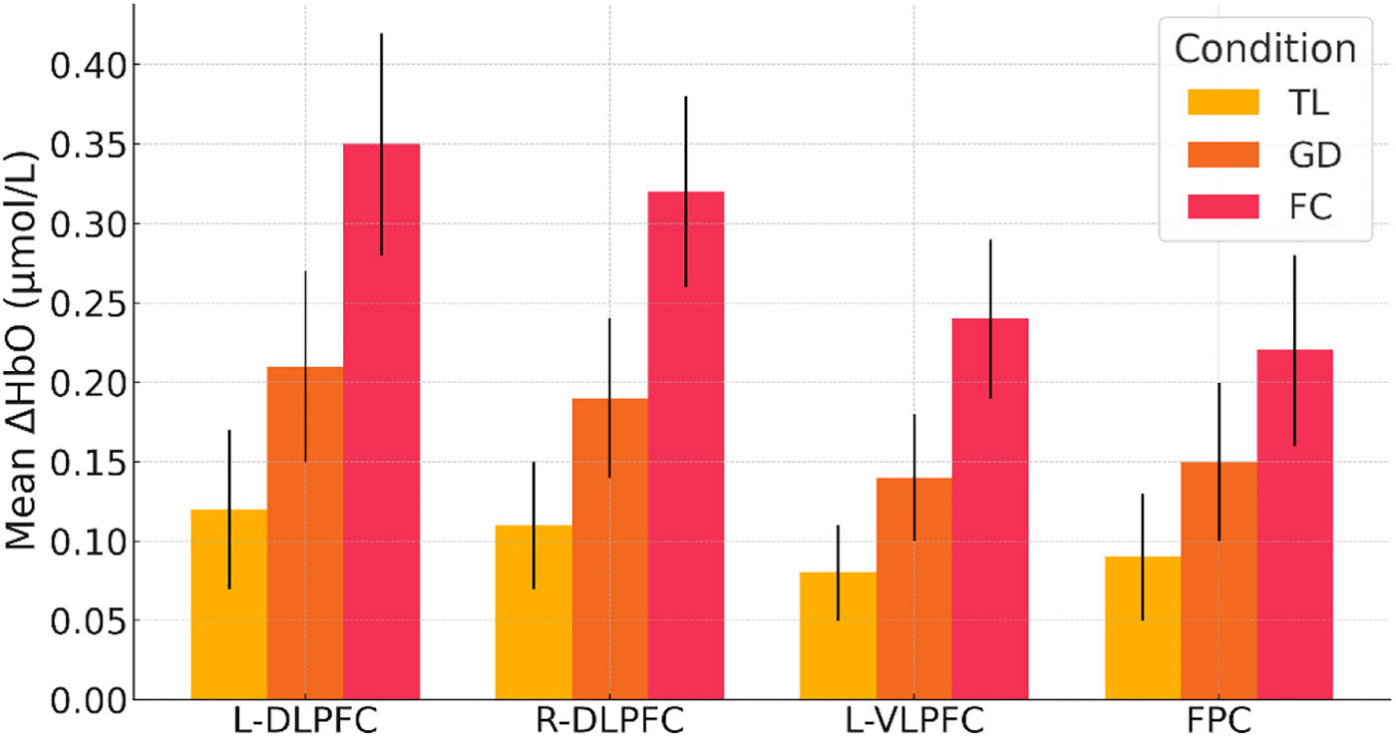
Mean prefrontal ΔHbO concentration by condition.

Analysis of hemodynamic responses revealed a significant main effect of instructional condition on mean ΔHbO in the L‐DLPFC (*F* (2, 58) = 25.73, *p* < 0.001, *η* ^2^_p_ = 0.47). Post hoc comparisons showed that the FC condition elicited the highest activation (0.35 ± 0.07 µmol/L), significantly greater than both the TL (0.12 ± 0.05 µmol/L; *p* < 0.001) and GD conditions (0.21 ± 0.06 µmol/L; *p* = 0.001). Similar patterns were observed in the right DLPFC (*F* (2, 58) = 23.94, *p* < 0.001, *η* ^2^_p_ = 0.45), the L‐VLPFC (*F* (2, 58) = 19.87, *p* < 0.001, *η* ^2^_p_ = 0.41), and the FPC (*F* (2, 58) = 14.52, *p* < 0.001, *η* ^2^_p_ = 0.33) (Table 4, Figure 3).

**TABLE 4:** Prefrontal ΔHbO concentration changes (µmol/L).

Region	TL	GD	FC	*F* (2, 58)	*p*	*η* ^2^_p_
L‐DLPFC	0.12 ± 0.05	0.21 ± 0.06	0.35 ± 0.07	25.73	< 0.001	0.47
R‐DLPFC	0.11 ± 0.04	0.19 ± 0.05	0.32 ± 0.06	23.94	< 0.001	0.45
L‐VLPFC	0.08 ± 0.03	0.14 ± 0.04	0.24 ± 0.05	19.87	< 0.001	0.41
FPC	0.09 ± 0.04	0.15 ± 0.05	0.22 ± 0.06	14.52	< 0.001	0.33

**FIGURE 4:**
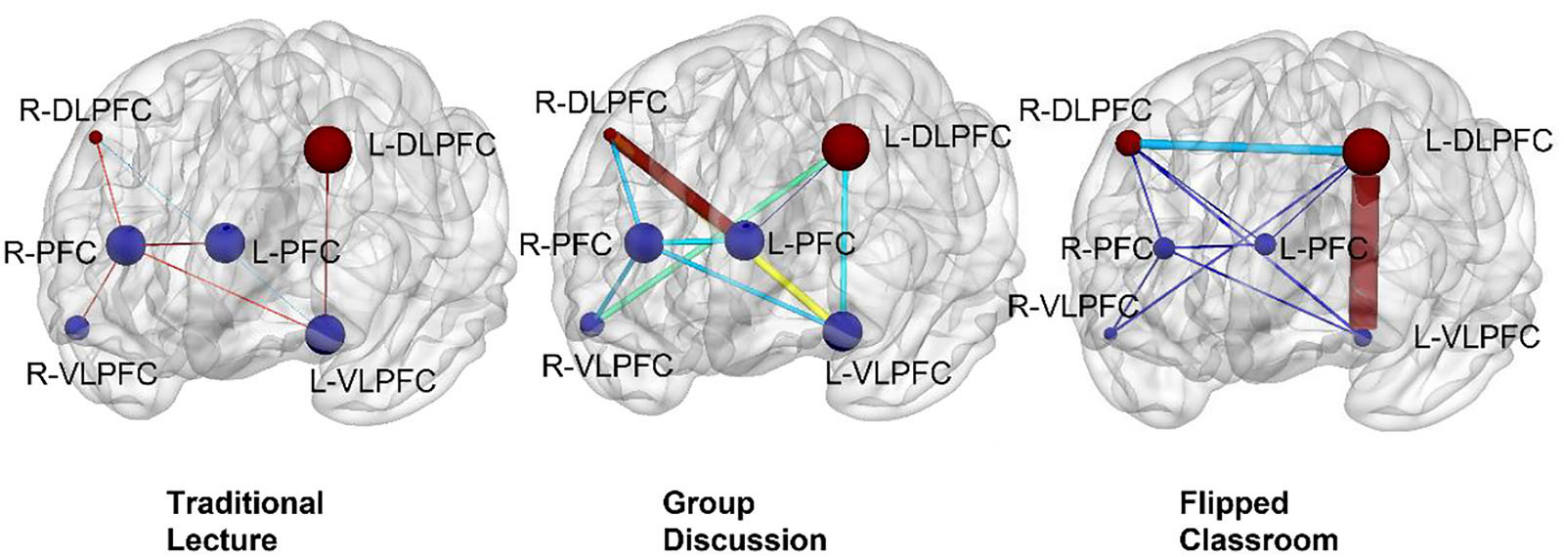
Prefrontal functional connectivity by condition.

Functional connectivity analysis showed a significant main effect of instructional condition on L‐DLPFC to L‐VLPFC connectivity strength (*F* (2, 58) = 17.85, *p* < 0.001, *η* ^2^_p_ = 0.38). The FC condition produced the strongest connectivity (FC = 0.48 ± 0.11), significantly higher than both the TL (0.29 ± 0.09; *p* < 0.001) and GD conditions (0.37 ± 0.10; *p* = 0.008). The GD condition also showed significantly stronger connectivity than the TL condition (*p* = 0.013), indicating enhanced prefrontal network coordination under more interactive instructional modes (Figure 4).

### 3.3 Brain–Behavior Correlations

Pearson correlation analysis revealed a significant positive relationship between L‐DLPFC activation and total comprehension score (*r* = 0.51, *p* < 0.001), with the strongest correlation found in the higher‐order semantic subscore (*r* = 0.68, *p* < 0.001). Furthermore, L‐DLPFC–L‐VLPFC connectivity strength was negatively correlated with modern‐meaning interference cost (*r* = −0.57, *p* = 0.001).

## 4 Discussion

This study used behavioral measurement and fNIRS to investigate the different effects of three teaching methods—TL, GD, and FC—on college students' cognitive understanding and prefrontal cortex activation. The findings provide consistent evidence from both behavioral performance and neural data, supporting the superiority of the FC method in promoting cognitive understanding, reducing cognitive load, and enhancing neural engagement in the prefrontal network.

The behavioral results of this study indicate that the FC performed optimally in the immediate comprehension test of college students' language courses, significantly outperforming TLs and GDs, with the most notable differences observed in the higher‐order semantic processing dimension (*η* ^2^_p_ = 0.42). This finding aligns with the expectations of cognitive load theory, which posits that FC reduces cognitive load in the classroom through pre‐class self‐directed learning, thereby freeing up more working memory resources for deep semantic integration (Sweller et al. 2019; Skulmowski and Xu 2022). Notably, while the GD group outperformed the TL group, its performance remained significantly below that of the FC group. This partially contradicts previous research on the effects of collaborative learning—suggesting that in disciplines like Chinese language education, which heavily rely on individual text close reading, structured knowledge internalization (such as FC's pre‐reading assessment mechanism) may be more effective in promoting concept mastery than mere interactive discussion (Wipawayangkool and Teng 2019). These findings have important implications for higher education: the advantages of FC extend beyond knowledge transmission efficiency, potentially enhancing students' critical reading abilities through optimized allocation of prefrontal cognitive resources (as subsequent fNIRS results will reveal in DLPFC activation patterns) (Diamond 2013).

This study found that GD performed best in semantic conflict tasks, with a significantly lower modern word meaning interference cost (152 ± 38 ms) than TLs and FCs, and the highest accuracy rate (86.2 ± 6.7%). This result suggests that GD may enhance students' ability to monitor and resolve semantic conflicts by promoting immediate verbal interaction and the exchange of perspectives, consistent with previous research on how collaborative learning enhances cognitive flexibility (Ng et al. 2025). Notably, while FC outperformed in overall comprehension tests, it lagged behind GD in semantic conflict tasks, suggesting that different teaching methods may have distinct advantages for different cognitive dimensions of language learning: FC is more conducive to systematic knowledge integration, while GD excels at cultivating real‐time semantic coordination skills. This finding has important implications for instructional design: in teaching scenarios requiring rapid semantic judgment (e.g., translating classical texts into modern Chinese), discussion‐based instruction should be prioritized, while in scenarios requiring in‐depth text analysis, the structured advantages of FC can be leveraged. Future research could explore blended teaching models to integrate the cognitive benefits of different methods (Lim et al. 2023; Raes 2022).

The subjective cognitive load analysis in this study indicates that the FC can significantly reduce learners' overall psychological load (NASA‐TLX total score *η* ^2^_p_ = 0.41), with the most prominent advantages in the dimensions of psychological needs (*η* ^2^_p_ = 0.39) and frustration (*η* ^2^_p_ = 0.43). This finding supports the hypothesis of the “distraction effect” in cognitive load theory (Craik 2014), suggesting that FC reduces cognitive pressure by moving the acquisition of foundational knowledge to an earlier stage, thereby alleviating the cognitive load associated with real‐time information processing in the classroom. Notably, although the GD group had significantly lower cognitive load than the TL group, it remained higher than the FC group. This contrasts interestingly with the GD group's advantage in the semantic conflict task in behavioral outcomes—suggesting that discussion‐based teaching, while enhancing specific cognitive abilities, may increase psychological burden due to factors like social pressure (van Son et al. 2020). These findings offer important insights for teaching practice: FC not only improves learning outcomes but also optimizes the learning experience, which has potential value for maintaining students' long‐term learning motivation. Future research could explore how to further balance the relationship between cognitive benefits and psychological load by adjusting the pre‐class task design of FC (Sims and Fletcher‐Wood 2021).

This study used fNIRS technology to reveal the differential effects of different teaching methods on neural activity in the prefrontal cortex. The findings indicate that the FC significantly enhances blood oxygen activity in the L‐DLPFC (ΔHbO = 0.35 ± 0.07 µmol/L) and promotes functional connectivity between the L‐DLPFC and the L‐VLPFC (FC = 0.48 ± 0.11). This finding provides a neurobiological explanation for the superior performance of FC in cognitive tests (Zhan et al. 2024). The high activation state of the L‐DLPFC suggests that FC may effectively mobilize working memory and higher‐order cognitive control resources through its structured learning design (Bao et al. 2022), while enhanced prefrontal functional connectivity reflects more efficient neural information integration processes (Liu et al. 2021). Notably, while GD also exhibited superior neural activation patterns compared to TL, its effect size was significantly smaller than that of FC, which may be related to GD's dispersed demand on cognitive resources. These findings provide direct evidence for understanding the neural basis of different teaching methods, suggesting that FC achieves dual improvements in cognitive outcomes and neural efficiency by optimizing the collaborative working patterns of the prefrontal network (Zhang et al. 2025). Educational practice can thus design more neuroscience‐informed teaching schemes, selecting the optimal teaching method based on different learning objectives (e.g., knowledge integration or semantic coordination).

This study revealed a direct association between prefrontal neural activity and language learning performance: the activation intensity of the L‐DLPFC was significantly positively correlated with the total comprehension score (*r* = 0.51) and the higher‐order semantic score (*r* = 0.68), while the functional connectivity intensity between the L‐DLPFC and the L‐VLPFC was significantly negatively correlated with the modern word meaning interference cost (*r* = −0.57). These findings provide the first validation of the “prefrontal cortex collaborative working hypothesis” in a real classroom setting—that is, the DLPFC is responsible for maintaining semantic working memory, while the VLPFC regulates conflict resolution, and their efficient coupling forms the neural basis for deep language learning (Friedman and Robbins 2022). Notably, higher‐order semantic processing showed the strongest correlation with neural indicators, providing objective neural evidence for “higher‐order thinking cultivation” in language teaching and suggesting that instructional design should prioritize enhancing functional integration of the prefrontal network (Molnar‐Szakacs and Uddin 2022). These brain–behavior association patterns not only offer new biomarkers for assessing instructional effectiveness but also lay the theoretical foundation for developing precision teaching strategies grounded in brain science evidence.

Several limitations of this study should be acknowledged. First, the relatively small sample size (*N* = 30), while appropriate for a within‐subject design, may limit the generalizability of findings across different cultural or educational contexts. Second, the exclusive recruitment of participants from a single university's Chinese language program restricts the external validity of our results for other academic disciplines or institutional settings. Third, the absence of long‐term follow‐up assessments precludes definitive conclusions about the durability of the observed teaching method effects on neural activation patterns. Finally, while fNIRS offers significant advantages for ecological classroom research, its technical limitations must be recognized—particularly its restriction to cortical surface measurements and inability to assess deeper brain structures that are known to contribute to learning processes.

Future research should expand sample sizes and include participants from diverse educational and cultural backgrounds to enhance generalizability. In addition, longitudinal studies are needed to examine the long‐term neural and cognitive effects of different teaching approaches. Combining fNIRS with other neuroimaging techniques (e.g., EEG) could also provide a more comprehensive understanding of brain activation dynamics during language learning.

## 5 Conclusion

In conclusion, this study demonstrates that the FC model is the most effective instructional strategy among the three tested, yielding superior comprehension outcomes, reduced cognitive load, and enhanced prefrontal activation and connectivity. Notably, the use of portable fNIRS sensors enabled real‐time, noninvasive monitoring of cortical dynamics in authentic learning contexts—bridging the gap between laboratory neuroscience and educational practice. This sensor‐based approach revealed that active learning environments not only improve behavioral performance but also promote deeper neural engagement, particularly within the dorsolateral and ventrolateral prefrontal networks. These findings underscore the utility of wearable neuroimaging sensors in evaluating and guiding evidence‐based instructional design, with the observed neural mechanisms likely generalizable to STEM disciplines and other humanities courses where higher‐order cognitive processing and prefrontal engagement are equally crucial.

## Author Contributions

All authors were involved in the experimental design and writing the manuscript for this study.

## Ethics Statement

Written informed consent was obtained from all participants prior to the experiment, and the study protocol was approved by Shanghai University of Political Science and Law's ethics review board (Approval ID: 10277SPSL2025).

## Consent

All participants and authors agreed to publish all aspects of this study.

## Conflicts of Interest

The authors declare no conflicts of interest.

## Peer Review

The peer review history for this article is available at https://publons.com/publon/10.1002/brb3.70934.

## Data Availability

All data from this study are in the manuscript; please contact the corresponding author if you need anything else.
